# Back from the brink: programmed cell revival for regeneration

**DOI:** 10.1038/s44318-025-00538-6

**Published:** 2025-08-21

**Authors:** Balamurugan Sundaram, Thirumala-Devi Kanneganti

**Affiliations:** Department of Immunology, St. Jude Children’s Research Hospital, Memphis, TN USA

**Keywords:** Autophagy & Cell Death, Signal Transduction, Stem Cells & Regenerative Medicine

## Abstract

Recent work in *The EMBO Journal* uncovers a new NF-ĸB-mediated process for dying cell recovery and tissue regeneration.

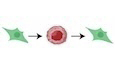

## Teaser

Activation of cell death pathways typically leads to the irreversible destruction of the cell. However, in this issue of *The EMBO Journal*, Dhar et al show that cells can recover from near-death states through an NF-ĸB-mediated programmed cell revival process that promotes tissue regeneration and restores function, revealing an unexpected link between cell death and tissue regeneration.

Regulated cell death pathways are genetically controlled processes with critical roles in development, homeostasis, immunity, and disease. These pathways are broadly classified into non-lytic and lytic forms. The canonical non-lytic pathway, apoptosis, is characterized by membrane blebbing that encapsulates the cellular contents, effectively preventing their release and mitigating downstream inflammation (Kerr et al, 1972). In contrast, lytic cell death pathways triggered by innate immune sensors (e.g., pyroptosis and PANoptosis) result in membrane rupture and cell lysis, which can release cytokines and damage-associated molecular patterns (DAMPs) to drive inflammation (Sundaram et al, 2024). Cell death plays a vital role in normal development and the maintenance of tissue homeostasis by eliminating unnecessary or damaged cells during embryogenesis and growth. Moreover, regulated cell death serves as a key component of the innate immune response, particularly through PANoptosis, to eliminate infected cells, reduce pathogen load, and prevent the spread of infection. However, excessive cell death can become pathogenic and promote tissue damage or inflammatory diseases (Kayagaki et al, 2024; Man and Kanneganti, 2024). Therefore, the activation of cell death pathways is tightly regulated to maintain the balance between health and disease.

Traditionally, cell death is considered an irreversible process – each cell that reaches the necessary threshold of cell death molecule activation will assemble multi-protein cell death complexes and inevitably proceed to the final execution of cell death. However, more recent studies have challenged this paradigm, showing that some forms of cell death can be reversed under certain conditions (Nano et al, 2023; Sun et al, 2017; Tang et al, 2012). Understanding the mechanisms of reversible cell death has significant therapeutic implications. For instance, promoting reversibility could reduce the pathogenicity of inflammatory diseases. Conversely, in cancer, reversible cell death may contribute to drug resistance, necessitating increases in drug dosing to improve treatment efficacy.

**Figure 1:**
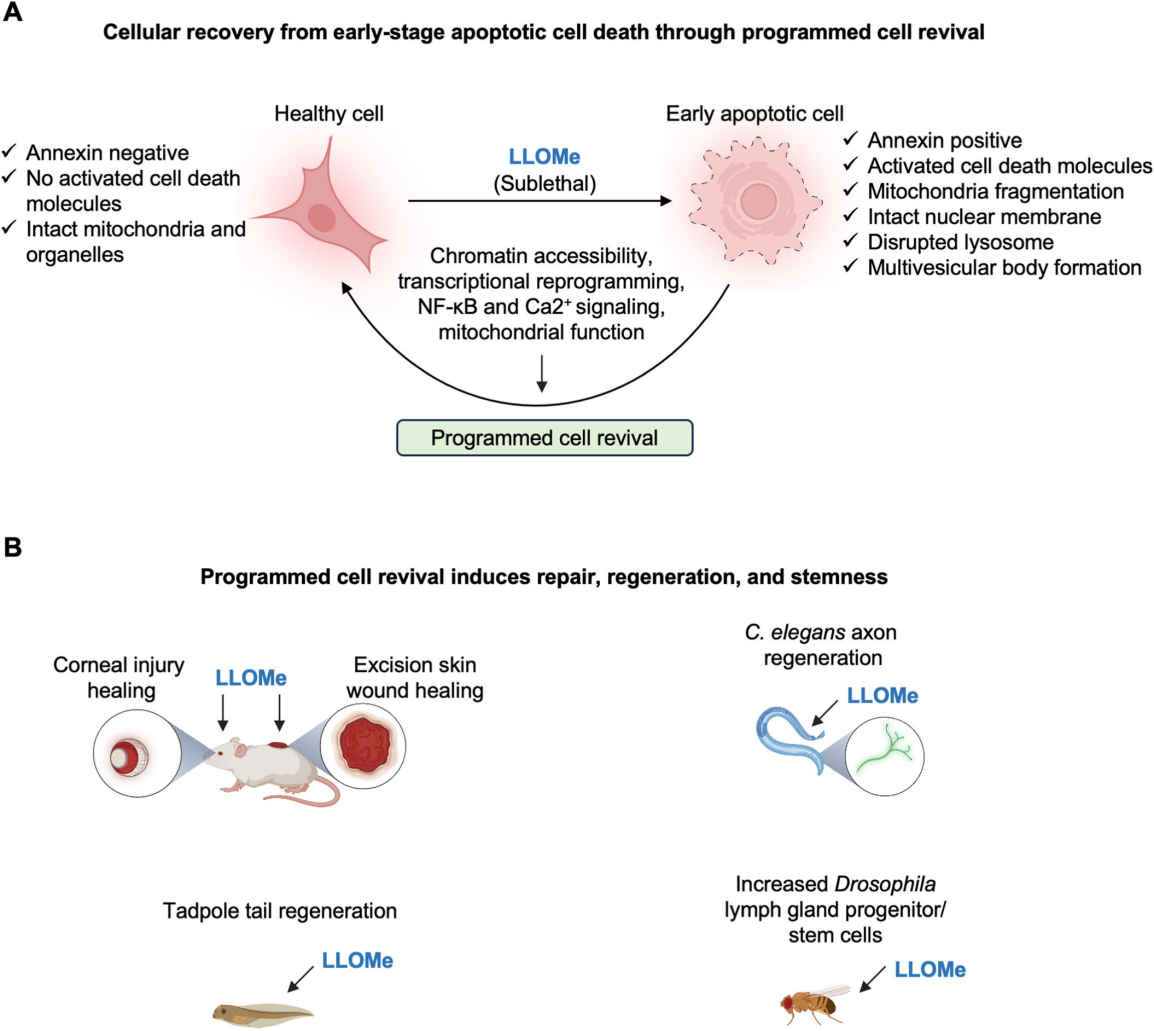
Inducing programmed cell revival promotes repair, regeneration, and stemness. In response to a sublethal dose of LLOMe, cells initiate apoptosis, but then undergo a revival mechanism mediated by NF-κB signaling, calcium (Ca^2+^) signaling, and mitochondrial function (**A**). This “programmed cell revival” promotes repair, regeneration, and stemness in multiple species (**B**). Figure was created using BioRender.

Therefore, in this issue of *The EMBO Journal*, Dhar et al sought to improve our understanding of the key molecular mechanisms that regulate the reversal of cell death and apply this knowledge to tissue repair (Dhar et al, 2025). In their study, the authors used a sublethal dose of the lysosomotropic agent L-Leucyl-L-leucine methyl ester (LLOMe) to induce apoptotic cell death (Johansson et al, 2010) in mouse embryonic fibroblasts (MEFs) and characterize the cell revival process. At the initial stage following LLOMe treatment, cells detach from the growth surface and display an apoptotic phenotype, suggesting they are undergoing cell death. However, at later stages, most of the floating cells reattach and regain their typical morphology, with a reduction in the activation of cell death molecules (Fig. 1A). These results indicate that cells can recover from the brink of cell death in response to LLOMe. This phenomenon occurs in multiple non-immune cell types, including primary MEFs and cardiac fibroblasts, as well as several cell lines from hamsters, mice, and humans (Dhar et al, 2025).

At the organellar level, shortly after treatment with LLOMe, microtubules, mitochondria, Golgi, and the endoplasmic reticulum are fragmented; however, these structures progressively recover within 2–3 h and return to near-normal morphology by 16 h post-treatment. Additionally, reviving cells display dramatic changes in endosomes, autophagosomes, and lysosomes, including the formation of abnormally large EEA1-positive early endosomes, LC3-positive autophagosomes, and Rab7/lysotracker-positive acidic vacuoles resembling multivesicular bodies. These large acidic compartments are enzymatically active and frequently surrounded by mitochondrial networks during revival, suggesting a role for metabolic support in driving the recovery. Mitochondrial membrane potential, which sharply declines after LLOMe treatment, rebounds within 2 h of reattachment and normalizes by 24 h post-treatment, correlating with reduced reactive oxygen species (ROS) levels and restored mitochondrial function; these features are key indicators of energy restoration and cell survival (Dhar et al, 2025). Together with the morphological features, these organellar changes support the concept that cells that have initiated a cell death process can reverse this and undergo “programmed cell revival” to return to their typical functional state.

Transcriptionally, this revival process is associated with a distinct cluster of genes that is temporally induced during the revival stages, further suggesting that revival from early apoptosis is a tightly regulated, or programmed, process. Gene ontology enrichment analysis in reviving cells identified a molecular signature resembling that of embryonic cells, indicating that the cells are likely reinitiating a new life program. Specifically, genes from signaling pathways with roles in embryonic development, cell growth and differentiation, stem cell proliferation and maintenance, tissue repair, and regeneration (Gumede et al, 2024) are upregulated upon initiation of the programmed cell revival process; these include *Nf-kb*, *Foxo*, *Runx1*, *Wnt*, *Notch1*, *p53*, *Nrf2*, *Tgf-β*, *Atf4*, *Npas4*, and *Mtor* (Dhar et al, 2025). Moreover, chromatin accessibility is significantly increased during the revival stage compared to the early apoptotic stage, and NF-κB-p65 is a critical transcription factor regulating genes with increased chromatin accessibility during revival. The central role of NF-κB was confirmed using both chemical and genetic approaches, providing a high level of confidence that NF-κB is critical in cell revival. Additionally, beyond chromatin accessibility and transcriptional regulation, Ca^2+^ signaling and mitochondrial function are also key regulators of the programmed cell revival process (Fig. 1A) (Dhar et al, 2025).

Based on this comprehensive characterization of programmed cell revival, several parallels can be seen between the pathways activated in programmed cell revival and wound healing. For example, FOXO, WNT, NOTCH, and TGF-β signaling pathways all have well-characterized roles in wound healing (Gumede et al, 2024). These same pathways are activated during programmed cell revival (Dhar et al, 2025), suggesting that programmed cell revival could be used as a strategy to promote wound healing. Indeed, treating mice with LLOMe to induce programmed cell revival improves skin wound healing and corneal repair. This paradigm can also be applied across species, as treatment with LLOMe improves the regeneration of amputated tails in tadpoles, enhances axonal regrowth in *Caenorhabditis elegans*, and increases hematopoietic stem cell production in *Drosophila melanogaster* (Fig. 1B) (Dhar et al, 2025). These in vivo results suggest that a sublethal dose of LLOMe could be tested clinically for tissue repair and wound healing.

These new insights into the molecular mechanisms of programmed cell revival have promising therapeutic implications, while also opening new directions for experimental investigation. For instance, it remains unclear what molecular signals cause a cell to choose revival over death, and whether these signals are conserved across different cell and tissue types. Additionally, while the programmed cell revival process is now comprehensively characterized in apoptotic cells, future work will be needed to determine whether a similar revival process is possible during innate immune or inflammatory cell death pathways to prevent the release of cytokines and DAMPs. As the release of even small amounts of cytokines or DAMPs can be sufficient to perpetuate broader inflammation and paracrine PANoptosis in bystander cells (Man and Kanneganti, 2024), defining a potential programmed cell revival process during inflammatory cell death would provide key insights into therapeutic strategies to mitigate this release.

While future research will be needed to address these questions, this work from Dhar et al presents a compelling, paradigm-shifting view of cell fate. By demonstrating that cell death signaling can transition into revival and regeneration, their work challenges long-standing assumptions and opens new avenues in cell and molecular biology, regenerative medicine, and therapeutic innovation to harness this revival program for disease treatment.
